# Efficiency of pulmonary nodule risk scoring systems in Turkish population

**DOI:** 10.1007/s13304-024-01901-8

**Published:** 2024-06-28

**Authors:** Hakan Nomenoğlu, Göktürk Fındık, Mehmet Çetin, Koray Aydoğdu, Selim Şakir Erkmen Gülhan, Pınar Bıçakçıoğlu

**Affiliations:** 1 Department of Thoracic Surgery, University of Health Sciences, Ankara Atatürk Sanatoryum Training and Research Hospital, Ankara, Turkey; 2 Department of Thoracic Surgery, Ministry of Health, Nigde Omer Halisdemir Training and Research Hospital, Nigde, Turkey; 3 Department of Thoracic Surgery, University of Health Sciences, Ankara Etlik City Hospital, Ankara, Turkey

**Keywords:** Pulmonary nodule, Lung cancer, Malignancy risk

## Abstract

Malignancy risk calculation models were developed using the clinical and radiological features. It was aimed to compare pulmonary nodule risk calculation models and evaluate their effectiveness and applicability for the Turkish population. Between 2014 and 2019, 351 patients who were operated on for pulmonary nodules were evaluated with the following data: age, gender, smoking history, family history of lung cancer, extrapulmonary malignancy and granulomatous disease, nodule diameter, attenuation character, side, localization, spiculation, nodule count, presence of pulmonary emphysema, FDG uptake in PET/CT of the nodule, and definitive pathology data. Malignancy risk scores were calculated using the equations of the Brock, Mayo, and Herder models. The results were evaluated statistically. The mean age of the 351 patients (236 men, 115 women) was 57.84 ± 10.87 (range 14–79) years, and 226 malignant and 125 benign nodules were observed. Significant correlations were found between malignancy and age (*p* < 0.001), nodule diameter (*p* < 0.001), gender (*p* < 0.009), speculation (*p* < 0.001), emphysema (*p* < 0.05), FDG uptake (*p* < 0.001). All three models were found effective in the differentiation (*p* < 0.001). The ideal threshold value was determined for the Brock (19.5%), Mayo (23.1%), and Herder (56%) models. All models were effective for nodules of > 10 mm, but none of them were for 0–10 mm. Brock was effective in ground**-**glass nodules (*p* = 0.02) and all models were effective for semi-solid and solid nodules. None of the groups could provide AUC values as high as those achieved in the original studies. This suggests the need to optimize models and malignancy risk thresholds for Turkish population.

## Introduction

The increase in the use of computed tomography (CT) has also increased the detection rate of incidental pulmonary nodules and related early stage lung cancers [1, 2]. To standardize the approach to these nodules, risk factors are determined according to the patients and the radiological characteristics of the nodules, and malignancy assessment and follow-up and treatment strategies are determined in light of different guidelines [3–6].

In addition, pulmonary nodule malignancy risk calculation models have been developed using the clinical features of patients and radiological features of nodules by thorax CT and PET–CT [7–9]. These models have been validated in the populations where the studies were conducted; in other societies, changes in efficacy may be observed depending on demographic characteristics or endemic diseases [10].

In this study, the clinical and radiological findings of patients who were operated on for pulmonary nodules and the effectiveness of the Brock University [7], Mayo Clinic [8], and Herder [9] malignancy risk calculation models developed with data from Western societies were evaluated using final pathology results. It was aimed to compare these models and evaluate their effectiveness and applicability for the Turkish population.

## Materials and methods

Local ethics committee approval was obtained for this study (No. 2012-KAEK-15/2195).

### Patient data

A total of 478 patients who were operated on for pulmonary nodules in our clinic between 2014 and 2019 were evaluated based on their digital information and archive files. The following data were obtained: age, gender, smoking history, family history of lung cancer, history of extrapulmonary malignancy, history of granulomatous disease, diameter of the nodule (largest diameter), attenuation character, side, lobe in which the nodule was located, presence of spiculation, total number of nodules detected, presence of pulmonary emphysema, fluorodeoxyglucose (FDG) uptake in positron emission tomography (PET) of the nodule, and definitive pathology data. Cases in which all data could not be reached, patients who had a history of lung or extrapulmonary malignancy within the last 5 years, patients who had endobronchial nodule components, and patients whose nodules were a result of metastasis were excluded. The remaining 351 patients were included in the study.

### Scoring systems

Pulmonary nodule malignancy scores were calculated using patient data in the equations of the Brock [7], Mayo [8], and Herder [9] models (Table 1). Although PET–CT is a semi-quantitative examination, FDG uptake in the nodules included in the study was scored as “no uptake,” “low uptake” (SUV_max_ < 2.5), “moderate uptake” (SUV_max_ 2.6–10), or “high uptake” (SUV_max_ > 10) as suggested by the Herder model according to a four-step scaling system [11] in line with nuclear medicine recommendation.

**Table 1:** Formulas of malignancy scoring models used for pulmonary nodules

Model	Formula	Probability of malignancy
Brock*	X = (0.0287 × (Age—62)) + Sex + Family History of Lung Cancer + Emphysema – (5.3854 × ((Nodule Diameter (mm)/10) – 0.5 – 1.58113883) + Nodule Type + Upper Lobe Localization – (0.0824 × (Nodule Count – 4)) + Spiculation – 6.7892	(e × / (1 + e ×))
*Sex: male: 0, female: 0.6011 Family History of Lung Cancer: present: 0.2961, absent: 0 Emphysema: present: 0.2953, absent: 0 Nodule Type: solid: 0, semi-solid: 0.377, ground-glass: -0.1276 Upper Lobe Localization: present: 0.6581, absent: 0 Spiculation: present: 0.7729, absent: 0		
Mayo**	x = − 6.8272 + (0.0391 × Age) + (0.7917 × History of Smoking) + (1.3388 × History of Extrathoracic Malignancy) + (0.1274 × Nodule Diameter (mm)) + (1.0407 × Spiculation) + (0.7838 × Upper Lobe Localization)	
**History of Smoking: present: 1, absent: 0 History of Extrathoracic Malignancy: present: 1, absent: 0 Spiculation: present: 1, absent: 0 Upper Lobe Localization: present: 1, absent: 0		
Herder	x = − 4.739 + 3.691 × (Percentage of Probability by the Mayo Clinic Model) + 0 (no uptake) + 2.322 (faint uptake) + 4.617 (medium uptake) + 4.771 (intense uptake)	

### Statistical analysis

All analyses were performed with IBM SPSS Statistics 21 (IBM Corp., Armonk, NY, USA). The conformity of the quantitative data to normal distribution was checked with histograms and Q–Q plots. Quantitative variables were summarized as mean ± standard deviation and median (minimum–maximum), while qualitative variables were given as frequency (percentage). Quantitative variables satisfying the assumption of normal distribution were analyzed with *t* tests in independent samples. Quantitative variables that did not satisfy the assumption of normal distribution were analyzed with the Mann–Whitney *U* test, and qualitative variables were analyzed with the Chi-square test. The success of the models in distinguishing between malignant and benign nodules was evaluated by receiver operating characteristic (ROC) curve analysis. The performance measures of sensitivity, specificity, correct classification rate, positive predictive value, and negative predictive value were calculated for cutoff points. Values of *p* < 0.05 ​​were considered statistically significant.

## Results

A total of 351 patients (236 men and 115 women) were included in the study. The mean age of these patients was 57.84 ± 10.87 (14–79) years, and 226 had malignant and 125 had benign nodules. Postoperative histopathological diagnoses are shown in Table 2.

**Table 2:** Postoperative histopathological diagnosis

Group			
Benign (*n* = 125) (%)		Malignant (*n* = 226) (%)	
Atypical adenomatous hyperplasia	1 (0.80)	Atypical carcinoid tumor	10 (4.42)
Abscess	1 (0.80)	Adenocarcinoma	139 (61.50)
Adenofibroma	1 (0.80)	Adenoid cystic carcinoma	1 (0.44)
Anthracotic lymph node	7 (5.60)	Adenosquamous carcinoma	7 (3.10)
Aspergillosis	1 (0.80)	Adenocarcinoma in situ	7 (3.10)
Arteriovenous malformation	1 (0.80)	Combined small cell carcinoma and adenocarcinoma	1 (0.44)
Fibrotic nodule	2 (1.60)	Large cell carcinoma	5 (2.21)
Fibrous tumor	1 (0.80)	Pleomorphic carcinoma	1 (0.44)
Granuloma	13 (10.40)	Squamous cell carcinoma	41 (18.14)
Hamartoma	46 (36.80)	Small cell carcinoma	1 (0.44)
Inflammation	5 (4.00)	Typical carcinoid tumor	13 (5.75)
Hydatid cyst	4 (3.20)		
Lymph node	1 (0.80)		
Mucous gland adenoma	2 (1.60)		
Necrosis	10 (8.00)		
Organized pneumonia	15 (12.00)		
Pneumoconiosis	1 (0.80)		
Sclerosing hemangioma	2 (1.60)		
Inflammatory pseudotumor	1 (0.80)		
Respiratory bronchiolitis	2 (1.60)		
Silicotic nodule	2 (1.60)		
Scar tissue	6 (4.80)		

Data are presented as frequency (percentage)

When 11 patients with a benign diagnosis (8.80%) and 18 patients with a malignant diagnosis (7.96%) with history of granulomatous disease were compared, no statistically significant difference was found (*p* = 0.944). In addition, there was no statistically significant relationship between history of granulomatous disease and number of detected nodules (*p* = 0.944) (Table 2).

When the quantitative data of individuals and nodules were evaluated according to groups, significant relationships were found between malignancy and age (*p* < 0.001) and nodule diameter (*p* < 0.001) (Table 3). When qualitative data were analyzed, gender (*p* < 0.009), nodule diameter (*p* < 0.05), spiculation (*p* < 0.001), emphysema (*p* < 0.05), and FDG uptake of the nodule (*p* < 0.001) were found to be significantly correlated with malignancy (Table 4).

**Table 3:** General characteristics of individuals and nodules by groups (quantitative data)

	Group	*n*	Mean ± std. dev	Median (min–max)	*p*
Age	Benign	125	52.21 ± 11.56	53 (14–73)	< 0.001^1^
	Malignant	226	60.96 ± 9.09	62 (26–79)	
Diameter (mm)	Benign	125	15.62 ± 5.88	15 (6–30)	< 0.001^1^
	Malignant	226	18.09 ± 5.91	18 (6–30)	
Nodule count	Benign	125	3.50 ± 2.80	3 (1–14)	0.607^2^
	Malignant	226	3.38 ± 2.72	2 (1–16)	

^1^*t* test in independent samples

^2^Mann–Whitney *U* test

**Table 4:** General characteristics of individuals and nodules by groups (qualitative data)

	Group		*p*
	Benign (*n* = 125) (%)	Malignant (*n* = 226) (%)	
Sex			
Male	73 (58.40)	163 (72.12)	0.009
Female	52 (41.60)	63 (27.88)	
History of smoking	76 (60.80)	155 (68.58)	0.141
Family history of lung cancer	15 (12.00)	40 (17.70)	0.210
History of extrathoracic malignancy	15 (12.00)	28 (12.39)	1.000
History of granulomatous disease	11 (8.80)	18 (7.96)	0.944
Nodule diameter			
0–10 mm	27 (21.60)	25 (11.06)	0.005
11–20 mm	73 (58.40)	127 (56.19)	
21–30 mm	25 (20.00)	74 (32.74)	
Nodule type			
Ground-glass	9 (7.20)	27 (11.95)	0.230
Semi-solid	70 (56.00)	131 (57.96)	
Solid	46 (36.80)	68 (30.09)	
Side			
Right	74 (59.20)	140 (61.95)	0.613
Left	51 (40.80)	86 (38.05)	
Lobe localization			
Lower	60 (48.00)	94 (41.59)	0.365
Middle	9 (7.20)	13 (5.75)	
Upper	56 (44.80)	119 (52.65)	
Spiculation	30 (24.00)	108 (47.79)	< 0.001
Emphysema	49 (39.20)	125 (55.31)	0.004
FDG uptake			
Absent	14 (11.20)	10 (4.42)	< 0.001
Faint	76 (60.80)	41 (18.14)	
Moderate	32 (25.60)	114 (50.44)	
Intense	3 (2.40)	61 (26.99)	

Data are summarized as frequency (percentage); *p* values were obtained using Chi-square tests

**Fig. 1:**
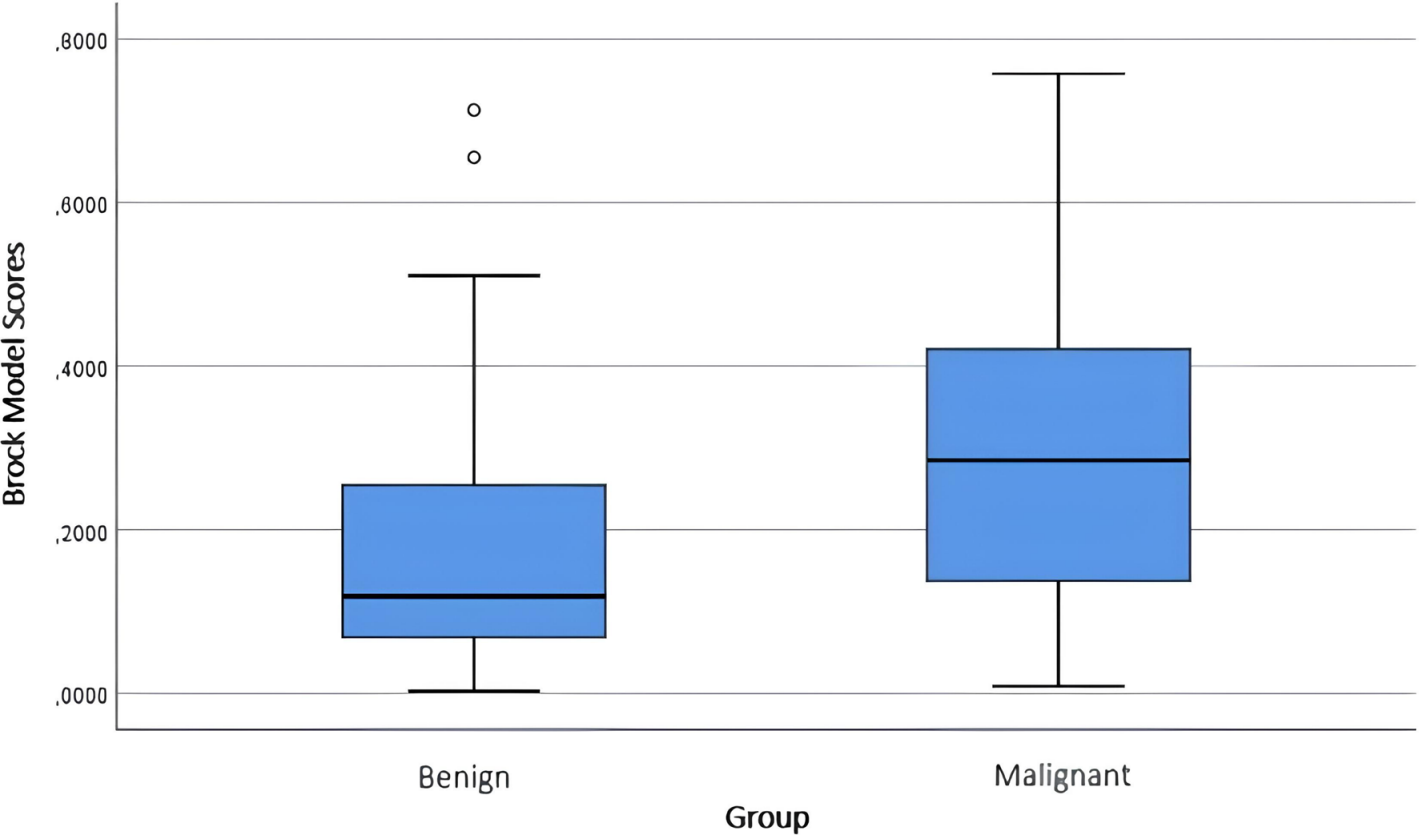
Score distribution of benign and malignant nodules according to the Brock model

**Fig. 2:**
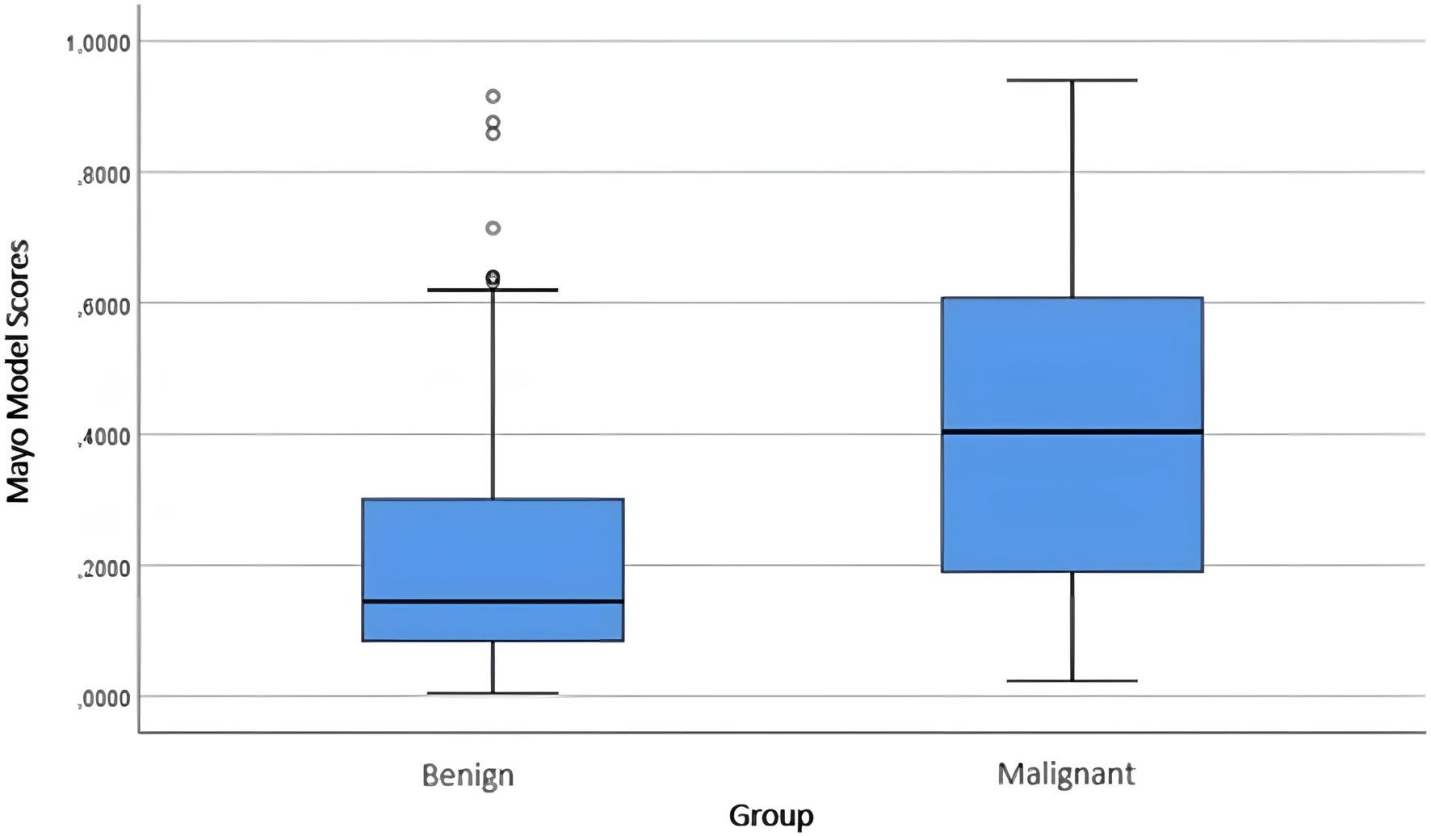
Score distribution of benign and malignant nodules according to the Mayo model

**Fig. 3:**
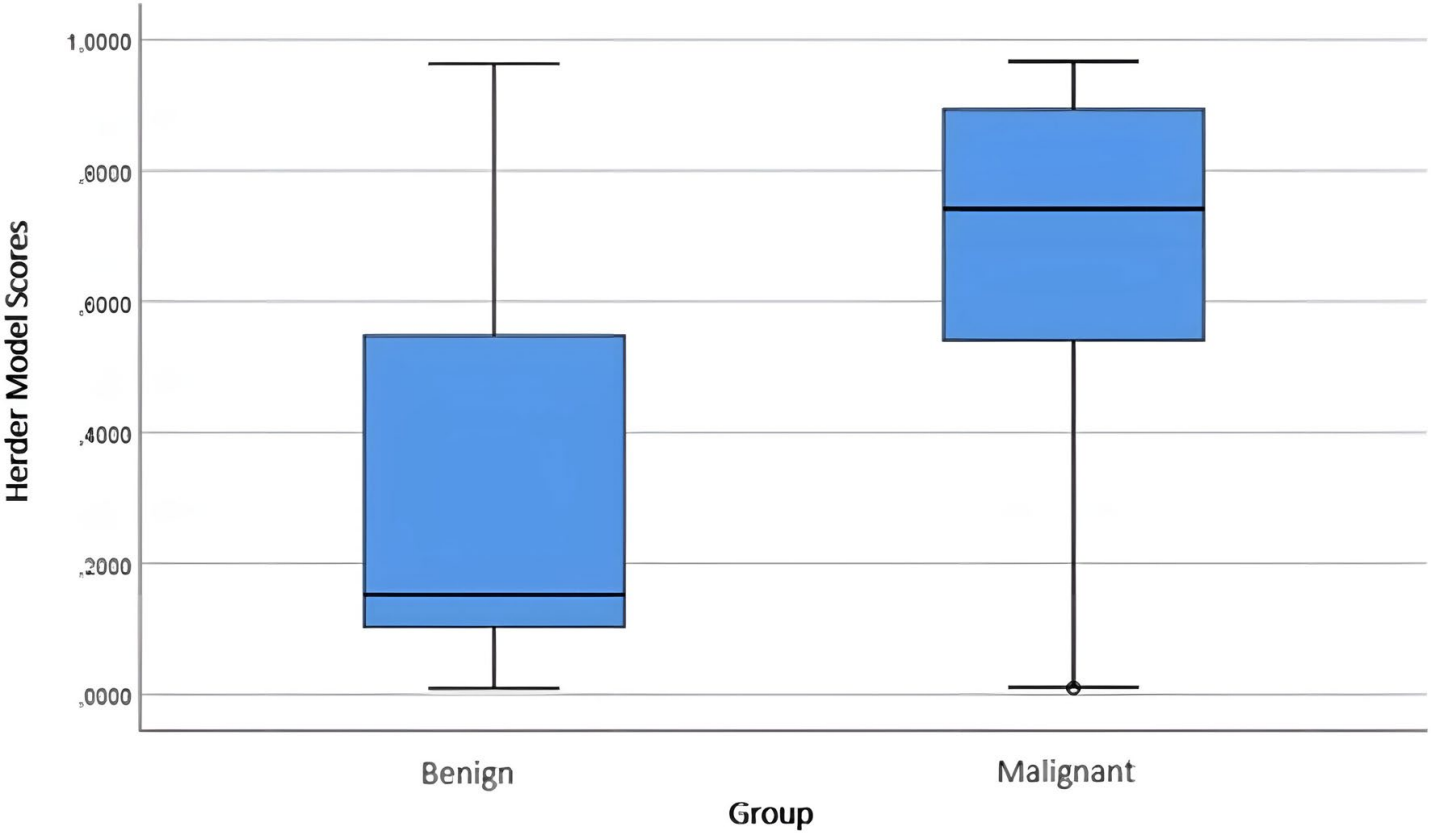
Score distribution of benign and malignant nodules according to the Herder model

When the scores for the pulmonary nodules obtained from the malignancy risk calculation models were evaluated, significant results were observed for all three models (*p* < 0.001) (Table 5). The score distributions of benign/malignant nodules according to the models are shown in Figs. 1, 2, and 3.

**Table 5:** Score data obtained from the models for benign and malignant nodules

	Group	*n*	Mean ± Std. Dev	Median (min–max)	*p*
Brock model	Benign	125	0.1699 ± 0.1434	0.1186 (0.0025–0.7129)	** < 0.001**
	Malignant	226	0.2940 ± 0.1886	0.2848 (0.0084–0.7576)	
Mayo model	Benign	125	0.2289 ± 0.2034	0.1446 (0.0046–0.9155)	** < 0.001**
	Malignant	226	0.4097 ± 0.2506	0.4033 (0.0229–0.9397)	
Herder model	Benign	125	0.3117 ± 0.2906	0.1521 (0.0089–0.9632)	** < 0.001**
	Malignant	226	0.6611 ± 0.2885	0.7417 (0.0094–0.9671)	

*p* values were obtained using the Mann–Whitney *U* test

While evaluating risk scores for the subcentimetric nodules, none of the calculation models showed significant performance result. (*p* = 0.654 for the Brock model, *p* = 0.898 for the Mayo model and *p* = 0.92 for the Herder model).

For nodules larger than 1 cm, the malignancy risk scores of the models were quite different compared to those for subcentimetric nodules. When nodules of 11–20 mm were examined, the risk scores of the models for benign and malignant nodules were compared and it was observed that the risk scores of malignant nodules were statistically significantly higher with all three models (*p* < 0.001 for all three models). For nodules of 21–30 mm, the malignancy probabilities obtained with the models were found to be statistically significantly higher for malignant nodules (*p* = 0.020 for the Brock model, *p* = 0.010 for the Mayo model, and *p* < 0.001 for the Herder model).

The efficacy of the models compared in this study was also evaluated according to the attenuation of the nodules by CT, and it was determined that nodules had different efficacy according to whether they were ground-glass, semi-solid, or solid nodules.

The malignancy probability scores obtained for ground-glass nodules were compared between malignant and benign nodules, and it was observed that the risk scores of malignant nodules were statistically significantly higher with the Brock model while there was no statistically significant difference between the risk scores of benign and malignant nodules with the Mayo and Herder models (*p* = 0.020 for the Brock model, *p* = 0.523 for the Mayo model, and *p* = 0.499 for the Herder model).

The scores observed from the models for semi-solid nodules were compared between benign and malignant nodules, and the probability of malignancy for malignant nodules was found to be statistically significantly higher with all three models (*p* < 0.001 for all three models).

Considering the efficacy of the models for solid nodules as the final attenuation character, the risk scores of the models for benign and malignant nodules were compared and it was seen that the malignancy risk values of malignant nodules were statistically significantly higher than those of benign nodules by all models (*p* < 0.001 for all three models).

**Fig. 4:**
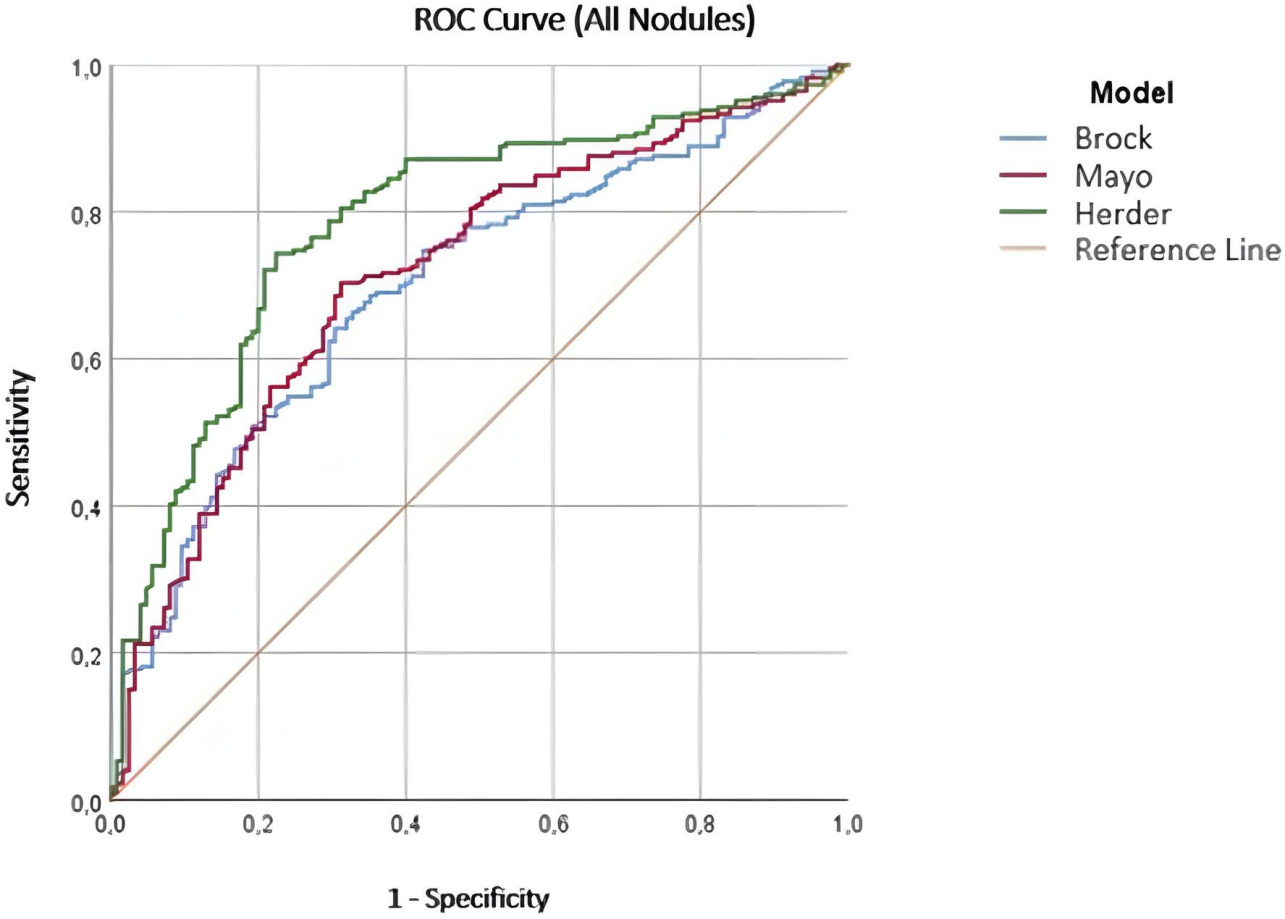
ROC curves for the Brock, Mayo and Herder models

To evaluate the effectiveness of the models in the differentiation of malignant and benign nodules, ROC curves were created and the area under the curve (AUC) was measured. The AUC value was 0.700 (95% CI 0.644–0.756) for the Brock model, 0.717 (95% CI 0.661–0.772) for the Mayo model, and 0.786 (95% CI 0.736–0.837) for the Herder model (Fig. 4).

While evaluating the performance of the models for subcentimetric nodules, the AUC values were found to be 0.536 for the Brock model, 0.490 for the Mayo model, and 0.508 for the Herder model.

When the ROC curves calculated to evaluate the performance of the malignancy risk estimation models for nodules larger than 1 cm in size were evaluated, the AUC value for nodules of 11–20 mm was found to be 0.724 for the Brock model, 0.755 for the Mayo model, and 0.841 for the Herder model. For nodules of 21–30 mm, the AUC values were 0.657 for the Brock model, 0.674 for the Mayo model, and 0.766 for the Herder model.

Areas under the ROC curves obtained from the risk scores of the models for ground-glass nodules were examined, the AUC values were 0.761 for the Brock model, 0.572 for the Mayo model, and 0.576 for the Herder model. The Brock model was superior to the Mayo and Herder models in the differentiation of benign and malignant ground-glass nodules.

When the AUC values of the models were examined for semi-solid nodules, they were found to be 0.741 for the Brock model, 0.740 for the Mayo model, and 0.797 for the Herder model.

While evaluating the performance of the models for solid nodules, it was seen that the AUC value was 0.719 for the Brock model, 0.758 for the Mayo model, and 0.891 for the Herder model.

To optimize the performance of the models for the 5% and 10% threshold values suggested in the guidelines according to the nodules in the population included in this study, to optimize the success of the benign/malignant distinction, separate sections were applied for the Brock (19.5%), Mayo (23.1%), and Herder (56%) models and ideal threshold values were determined [3, 5, 6] (Table 6).

**Table 6:** Performance measures for malignant/benign distinctions of the models

	Threshold Value	Sensitivity %	Specificity %	CCR %	PPV %	NPV %	AUC (95% CI)	*p*
Brock model	≥ 0.05	90.71	16.80	64.39	66.34	50.00	0.700 (0.644–0.756)	** < 0.001**
	≥ 0.10	81.42	40.00	66.67	71.04	54.35		
	≥ 0.195	64.16	69.60	66.10	79.23	51.79		
Mayo model	≥ 0.05	94.69	11.20	64.96	65.85	53.85	0.717 (0.661–0.772)	** < 0.001**
	≥ 0.10	87.61	32.80	68.09	70.21	59.42		
	≥ 0.231	70.35	68.80	69.80	80.30	56.21		
Herder model	≥ 0.05	95.58	11.20	65.53	66.06	58.33	0.786 (0.736–0.837)	** < 0.001**
	≥ 0.10	92.92	22.40	67.81	68.40	63.64		
	≥ 0.560	73.89	77.60	75.21	85.64	62.18		

*CCR* Correct classification rate, *PPV* Positive predictive value, *NPV* Negative predictive value, *AUC* Area under the curve, *CI* Confidence interval

*p* values were obtained for hypothesis H0: AUC = 0.5

The performances of the models were also evaluated according to nodule size, and it was seen that no model achieved a statistically significant difference for nodules of 0–10 mm, while all models were found to be effective for nodules of 11–20 mm and 21–30 mm (Table 7). The performance measures of the models according to nodule size are shown in Table 8.

**Table 7:** Nodule size scores from the models

	Diameter (mm)	Group	*n*	Mean ± std. dev	Median (min–max)	*p*
Brock model	0–10	Benign	27	0.0545 ± 0.0686	0.0314 (0.0025–0.3559)	0.654
		Malignant	25	0.0459 ± 0.0267	0.0456 (0.0084–0.1214)	
	11–20	Benign	73	0.1524 ± 0.1020	0.1181 (0.0268–0.4909)	** < 0.001**
		Malignant	127	0.2621 ± 0.1543	0.2336 (0.0241–0.6846)	
	21–30	Benign	25	0.3455 ± 0.1479	0.3272 (0.1209–0.7129)	0.020
		Malignant	74	0.4327 ± 0.1609	0.4150 (0.1542–0.7576)	
Mayo model	0–10	Benign	27	0.1378 ± 0.1160	0.1008 (0.0046–0.5475)	0.898
		Malignant	25	0.1278 ± 0.0949	0.0951 (0.0229–0.3698)	
	11–20	Benign	73	0.1751 ± 0.1459	0.1189 (0.0250–0.7142)	** < 0.001**
		Malignant	127	0.3482 ± 0.2144	0.2916 (0.0264–0.9077)	
	21–30	Benign	25	0.4844 ± 0.2218	0.5035 (0.0932–0.9155)	0.010
		Malignant	74	0.6104 ± 0.1916	0.6218 (0.1560–0.9397)	
Herder model	0–10	Benign	27	0.1803 ± 0.2208	0.1248 (0.0089–0.8698)	0.920
		Malignant	25	0.2607 ± 0.2868	0.1080 (0.0094–0.7761)	
	11–20	Benign	73	0.2624 ± 0.2555	0.1253 (0.0104–0.9012)	** < 0.001**
		Malignant	127	0.6467 ± 0.2533	0.6881 (0.0096–0.9671)	
	21–30	Benign	25	0.5976 ± 0.2754	0.7071 (0.1237–0.9632)	** < 0.001**
		Malignant	74	0.8212 ± 0.1938	0.8959 (0.2075–0.9670)	

*p* values were obtained using the Mann–Whitney *U* test

**Table 8:** Performance measures of models in the distinction of malignant and benign according to nodule size

	Nodule diameter (mm)	Threshold value	Sensitivity %	Specificity %	CCR %	PPV %	NPV %	AUC (95% CI)	*p*
Brock model	0–10	≥ 0.05	40.00	59.26	50.00	47.62	51.61	0.536 (0.375–0.697)	0.654
		≥ 0.10	4.00	88.89	48.08	25.00	50.00		
		≥ 0.195	0.00	96.30	50.00	0.00	50.98		
	11–20	≥ 0.05	95.28	6.85	63.00	64.02	45.45	0.724 (0.653–0.795)	** < 0.001**
		≥ 0.10	85.83	35.62	67.50	69.87	59.09		
		≥ 0.195	59.06	79.45	66.50	83.33	52.73		
	21–30	≥ 0.05	100.00	0.00	74.75	74.75	N/A	0.657 (0.535–0.778)	0.020
		≥ 0.10	100.00	0.00	74.75	74.75	N/A		
		≥ 0.195	94.59	12.00	73.74	76.09	42.86		
Mayo model	0–10	≥ 0.05	80.00	22.22	50.00	48.78	54.55	0.490 (0.330–0.649)	0.898
		≥ 0.10	44.00	48.15	46.15	44.00	48.15		
		≥ 0.231	16.00	81.48	50.00	44.44	51.16		
	11–20	≥ 0.05	94.49	10.96	64.00	64.86	53.33	0.755 (0.687–0.824)	** < 0.001**
		≥ 0.10	88.98	36.99	70.00	71.07	65.85		
		≥ 0.231	66.14	80.82	71.50	85.71	57.84		
	21–30	≥ 0.05	100.00	0.00	74.75	74.75	N/A	0.674 (0.548–0.800)	0.010
		≥ 0.10	100.00	4.00	75.76	75.51	100.00		
		≥ 0.231	95.95	20.00	76.77	78.02	62.50		
Herder model	0–10	≥ 0.05	64.00	25.93	44.23	44.44	43.75	0.508 (0.346–0.671)	0.920
		≥ 0.10	56.00	40.74	48.08	46.67	50.00		
		≥ 0.560	24.00	92.59	59.62	75.00	56.82		
	11–20	≥ 0.05	99.21	9.59	66.50	65.63	87.50	0.841 (0.783–0.900)	** < 0.001**
		≥ 0.10	96.06	23.29	69.50	68.54	77.27		
		≥ 0.560	76.38	82.19	78.50	88.18	66.67		
	21–30	≥ 0.05	100.00	0.00	74.75	74.75	N/A	0.766 (0.654–0.878)	** < 0.001**
		≥ 0.10	100.00	0.00	74.75	74.75	N/A		
		≥ 0.560	86.49	48.00	76.77	83.12	54.55		

*CCR* Correct classification rate, *PPV* Positive predictive value, *NPV* Negative predictive value, *AUC* Area under the curve, *CI* Confidence interval

*p* values were obtained for hypothesis H0: AUC = 0.5

The performances of the models were evaluated separately for ground-glass, semi-solid, and solid nodules according to the attenuation of the nodules. Statistical significance was observed for ground-glass nodules only with the Brock model (*p* = 0.02), while statistical significance was found for all models in the evaluation of semi-solid and solid nodules (Table 9). The performance measures of the models according to nodule attenuation are shown in Table 10.

**Table 9:** Nodule attenuation scores from the models

	Nodule attenuation	Group	*n*	Mean ± std. dev	Median (min–max)	*p*
Brock model	Ground-glass	Benign	9	0.0265 ± 0.0183	0.0197 (0.0084–0.0586)	0.020
		Malignant	27	0.1016 ± 0.1282	0.0456 (0.0084–0.5214)	
	Semi-solid	Benign	70	0.2056 ± 0.1600	0.1521 (0.0097–0.7129)	** < 0.001**
		Malignant	131	0.3587 ± 0.1867	0.3373 (0.0429–0.7576)	
	Solid	Benign	46	0.1435 ± 0.1025	0.1052 (0.0025–0.4467)	** < 0.001**
		Malignant	68	0.2458 ± 0.1411	0.2259 (0.0310–0.6234)	
Mayo model	Ground-glass	Benign	9	0.1198 ± 0.0960	0.0684 (0.0081–0.2833)	0.523
		Malignant	27	0.1766 ± 0.1697	0.1110 (0.0229–0.5839)	
	Semi-solid	Benign	70	0.2538 ± 0.2167	0.1835 (0.0337–0.8762)	** < 0.001**
		Malignant	131	0.4541 ± 0.2430	0.4846 (0.0375–0.9077)	
	Solid	Benign	46	0.2124 ± 0.1917	0.1386 (0.0046–0.9155)	** < 0.001**
		Malignant	68	0.4166 ± 0.2438	0.4040 (0.0319–0.9397)	
Herder model	Ground-glass	Benign	9	0.1869 ± 0.2567	0.0990 (0.0089–0.7158)	0.499
		Malignant	27	0.2655 ± 0.2980	0.1080 (0.0094–0.8934)	
	Semi-solid	Benign	70	0.3543 ± 0.3196	0.1635 (0.0104–0.9632)	** < 0.001**
		Malignant	131	0.7128 ± 0.2464	0.7991 (0.0140–0.9671)	
	Solid	Benign	46	0.2713 ± 0.2383	0.1377 (0.0099–0.8259)	** < 0.001**
		Malignant	68	0.7188 ± 0.2358	0.7798 (0.0271–0.9660)	

*p* values were obtained using the Mann–Whitney *U* test

**Table 10:** Performance measures of models in the distinction of malignant and benign according to nodule attenuation

	Nodule Attenuation	Threshold value	Sensitivity %	Specificity %	CCR %	PPV %	NPV %	AUC (95% CI)	*p*
Brock model	Ground-glass	≥ 0.05	48.15	77.78	55.56	86.67	33.33	0.761 (0.594–0.929)	0.020
		≥ 0.10	29.63	100.00	47.22	100.00	32.14		
		≥ 0.195	14.81	100.00	36.11	100.00	28.13		
	Semi-solid	≥ 0.05	99.24	8.57	67.66	67.01	85.71	0.741 (0.669–0.813)	** < 0.001**
		≥ 0.10	92.37	30.00	70.65	71.18	67.74		
		≥ 0.195	76.34	62.86	71.64	79.37	58.67		
	Solid	≥ 0.05	91.18	17.39	61.40	62.00	57.14	0.719 (0.625–0.812)	** < 0.001**
		≥ 0.10	80.88	43.48	65.79	67.90	60.61		
		≥ 0.195	60.29	73.91	65.79	77.36	55.74		
Mayo model	Ground-glass	≥ 0.05	77.78	22.22	63.89	75.00	25.00	0.572 (0.358–0.786)	0.523
		≥ 0.10	51.85	55.56	52.78	77.78	27.78		
		≥ 0.231	25.93	88.89	41.67	87.50	28.57		
	Semi-solid	≥ 0.05	96.95	10.00	66.67	66.84	63.64	0.740 (0.667–0.813)	** < 0.001**
		≥ 0.10	93.13	31.43	71.64	71.76	70.97		
		≥ 0.231	77.86	64.29	73.13	80.31	60.81		
	Solid	≥ 0.05	97.06	10.87	62.28	61.68	71.43	0.758 (0.668–0.848)	** < 0.001**
		≥ 0.10	91.18	30.43	66.67	65.96	70.00		
		≥ 0.231	73.53	71.74	72.81	79.37	64.71		
Herder model	Ground-glass	≥ 0.05	70.37	44.44	63.89	79.17	33.33	0.576 (0.358–0.794)	0.499
		≥ 0.10	55.56	55.56	55.56	78.95	29.41		
		≥ 0.560	25.93	88.89	41.67	87.50	28.57		
	Semi-solid	≥ 0.05	99.24	11.43	68.66	67.71	88.89	0.797 (0.729–0.866)	** < 0.001**
		≥ 0.10	97.71	22.86	71.64	70.33	84.21		
		≥ 0.560	80.15	70.00	76.62	83.33	65.33		
	Solid	≥ 0.05	99.24	11.43	68.66	67.71	88.89	0.797 (0.729–0.866)	** < 0.001**
		≥ 0.10	97.71	22.86	71.64	70.33	84.21		
		≥ 0.560	80.15	70.00	76.62	83.33	65.33		

*CCR* Correct classification rate, *PPV* Positive predictive value, *NPV* Negative predictive value, *AUC* Area under the curve, *CI* Confidence interval

*p* values were obtained for hypothesis *H0*: AUC = 0.5

## Discussion

When evaluated independently of other variables, it was seen that all scoring systems gave significant results in the differentiation of benign and malignant nodules. In addition, no significant results were observed for any scoring system with nodule sizes of < 1 cm, while all scoring systems were successful in the differentiation of benign and malignant nodules of > 1 cm.

According to nodule attenuation, while no scoring system gave significant results for ground-glass nodules, all scoring systems provided significant results for solid and semi-solid nodules.

In our study, there were significant relationships between age, nodule diameter, gender, spiculation, emphysema, and FDG uptake of the nodule and malignancy, which are among the parameters considered in these models, but no significant relationship was found between the other variables and malignancy.

When the ideal threshold values and different threshold values determined for each scoring system for our cases were evaluated, the obtained significance levels did not change. It was also observed that the history of granulomatous disease did not cause a significant change in the number of nodules.

When benign and malignant cases were compared according to the scores obtained from the pulmonary nodule malignancy prediction models evaluated in this study, the risk scores of malignant cases were found to be statistically significantly higher with all three models. However, when the mean malignancy risk score of benign nodules was considered, it was seen that it was 16.99% for the Brock model, 22.89% for the Mayo model, and 31.17% for the Herder model. The relevant threshold values attributed to malignancy risk probability for nodules in terms of benign/malignant distinction were determined as 5% in the ACCP and Fleichner guidelines and as 10% in the BTS guidelines [3, 5, 6]. Therefore, most of the nodules in our study had risk scores above the specified threshold values. In our opinion, the reason for this is that almost all of the nodules in the cases included in this study were operated on in our clinic due to moderate or high suspicion of malignancy. To optimally distinguish between benign and malignant nodules in patients with high mean risk scores by all three models and with many clinical and radiological risk factors, for the models evaluated in this study, it was necessary to determine new threshold values for the possibility of malignancy as specified in the guidelines. As a result of the statistical analyses, optimal threshold values were found to be 19.5% for the Brock model, 23.1% for the Mayo model, and 56% for the Herder model. Although the new threshold values slightly decreased the overall sensitivity of the models in distinguishing benign and malignant nodules, they had positive effects on other parameters, especially specificity and positive predictive values.

AUC for ROC curves were measured for evaluating the performances of the calculation models. It was observed that the Herder model performed significantly better than the Brock and Mayo models, which had very close AUC values. In light of this situation, FDG uptake in PET–CT may play an important role in the evaluation of pulmonary nodules. While developing the Herder model, only the performance of FDG uptake in the differentiation of benign and malignant nodules was examined, and no statistically significant difference was found for the performance of the Mayo model as used in this study. However, after integrating the FDG uptake of the nodule into the Mayo model, it was seen that the final version of the Herder model was statistically significantly superior to the performance of the Mayo model and isolated FDG uptake [9]. As can be understood here, the FDG uptake level of a nodule in PET–CT is not a sufficient parameter for evaluating the possibility of the malignancy of that nodule. However, when PET–CT findings are evaluated together with other clinical and radiological features of the patient, it becomes a valuable tool in determining the possibility of malignancy.

In addition, in our study, it was observed that the AUC values for all three models were lower than the AUC values reported in the original publications on the models’ development and validation (0.96 for the Brock model, 0.79 for the Mayo model, and 0.92 for the Herder model) [7–9]. This may be because, in addition to many other factors, almost all of the patients included in this study from our clinic were being followed due to a relatively high risk of malignancy. Therefore, the scores of malignant and benign nodules were generally closer to each other than they were in the populations studied in the original development of the models.

Compared to the AUC values obtained when all nodules were included, significant performance loss was detected upon differentiating benign and malignant nodules in all three models for subcentimetric nodules. There could be several reasons for this. First of all, the numbers of benign and malignant nodules included in this study were very close to each other (27 benign, 25 malignant). In the populations in which the models were developed, the malignancy rate was below 5% in both cohorts for the Brock model, 23% for the Mayo model, and 57% for the Herder model [7–9]. The performance degradation of the models may be due to this. Furthermore, while developing the Mayo model, all of the evaluated nodules were detected by chest X-ray [8]. Since subcentimetric nodules are more difficult to detect by chest X-ray than large nodules, the characteristic features of the detected subcentimetric nodules may have differed from our study. The same reasoning applies for the Herder model, since parameters other than PET–CT findings are calculated in the Herder model in contrast to the Mayo model. In addition, since none of the subcentimetric nodules in our study had moderate or high uptake of FDG, the guiding effect of PET–CT was limited, and the effectiveness of the Herder model may have therefore decreased. In the BTS guidelines, in accordance with the inferences to be made from the results of this study, the use of the Herder model is not recommended for nodules smaller than 8 mm [5]. Since all of the nodules included in the original study were detected by CT for the Brock model, the rate of subcentimetric nodules was higher than that evaluated by the other models [7]. Although a statistically significant effect was not observed, we think that the higher AUC value obtained for the Brock model compared to the other models was related to this. However, in our clinic, many patients with malignant subcentimetric nodules that should be conservatively followed-up according to the guidelines or even removed from follow-up were operated on thanks to the individual experiences and initiatives of the experienced radiologists and clinicians in our hospital, and these patients obtained curative treatment at the earliest possible stage. Sometimes clinicians or radiologists with quite experience may use clinical judgment which is different from the calculation model or guideline, and this is as effective as risk prediction models because of considering more variables and old experiences [12]. In addition, it is difficult to detect these nodules intraoperatively as well as in follow-up. Marking methods can also be used preoperatively [13].

For nodules larger than 1 cm, results were all statistically significant for all three models, both for solid and semi-solid nodules. In our opinion, with the elimination of the disadvantages of subcentimetric nodules, the malignancy risk estimation models achieved significant success in distinguishing between benign and malignant nodules. In addition, since nodules larger than 1 cm do not pose the difficulties for diagnostic factors that are seen with subcentimetric nodules, the AUC values were significantly higher.

The reason why the AUC values obtained for nodules of 11–20 mm were higher than those obtained for nodules of 21–30 mm, in our opinion, is the false positivity of large benign nodules. While the mean malignancy probabilities observed from the models for benign nodules of 11–20 mm were calculated as 15.24%, 17.51%, and 26.24% for the Brock, Mayo, and Herder models, respectively, these probabilities were calculated as 34.55%, 48.44%, and 59.66% for nodules of 21–30 mm. In other words, the mean probability of the malignancy of benign nodules of 21–30 mm in all models is higher than the optimal threshold values calculated for those models. This increases the false positive results and causes a negative effect on the performance measures of the nodules. Among the three models, the highest AUC value was obtained for the Herder model for both size ranges, and the lowest AUC value was that of the Brock model. However, no significant difference was observed between the AUC values of the Brock and Mayo models. Considering this finding, PET–CT is an important tool in the management of nodules of > 1 cm.

The efficacy of the models compared in this study was also compared according to the attenuation and the malignancy probability scores obtained for ground-glass nodules showed that only the Brock model determined malignant and benign nodules sufficiently. Ground-glass nodules are very difficult to evaluate, similar to subcentimetric nodules. In our study, it was an expected finding that the Mayo and Herder models, which could not make optimum use of these two factors, could not make effective distinctions between benign and malignant nodules, since spiculation, which has a significant difference between benign and malignant nodules, was not seen in ground-glass nodules due to their structures and generally low FDG avidity.

While AUC measurements for ground-glass nodules compared, in our opinion, the reason for the poor performance of the Mayo and Herder models in this regard may be that, similar to subcentimetric nodules, the nodules in the population included in the development of the Mayo model were evaluated after chest radiographs were reviewed [8]. Since ground-glass nodules, and especially those that are small in size, are difficult to detect on chest radiographs, the rate of ground-glass nodules included in the original study is likely very low compared to our study. Since the parameters of the Herder model, excluding PET–CT findings, are based on the Mayo model, the same problem is likely to be experienced with the Herder model. The Brock model, on the other hand, was created based on nodules detected by CT and the attenuation of the nodules was integrated into the model [7]. However, in the Brock model, the ground-glass character was a factor that reduced the possibility of malignancy, while 75% of the ground-glass nodules in our study were found to be malignant. Despite this, it is an interesting finding that the Brock model yielded the highest AUC value for ground-glass nodules among all groups evaluated by the Brock model in this study.

All three models successfully differentiated malignant and benign semi-solid nodules. Most of the semi-solid nodules included in this study were over 1 cm in size and it is possible that all of the models produced significant results in the differentiation of benign and malignant semi-solid nodules for this reason, in contrast to ground-glass nodules. In addition, when the mean malignancy probabilities of benign and malignant semi-solid nodules by the Brock model were examined, it was seen that they were higher than those obtained for solid and ground-glass nodules. This, in line with the model, suggests that the semi-solid nature of a nodule increases the possibility of malignancy.

When the AUC values of the models for semi-solid nodules were considered, excluding the Brock model, a significant increase was found in the AUC values of the other two models. In our opinion, the reason for this is likely related to the fact that the Mayo and Herder models are models developed based on nodules detected by chest radiography, as mentioned above while discussing the AUC values of the models for ground-glass nodules [8, 9]. The Mayo and Herder models may have been more successful in distinguishing benign and malignant semi-solid nodules compared to ground-glass nodules because the solid components of these nodules are increased. Therefore, the probability of their detection by chest X-ray also increases. In addition, it seems likely that the higher FDG uptake of semi-solid nodules compared to ground-glass nodules in this study contributed to the increased efficiency of the Herder model. The Herder model had the best performance among the three models for semi-solid nodules.

When it comes to solid nodules, all three models also differentiated malignant and benign ones successfully. Approximately 80% of the nodules in the two cohorts included in the original study for the development of the Brock model were solid nodules. Since the Mayo model and the Herder model, which is a derivation of the Mayo model, are models developed on the basis of nodules seen by chest X-rays, it is highly likely that the majority of the nodules included in those studies were solid. Models developed in studies in which solid nodules were the majority may have differentiated benign and malignant solid nodules more effectively in our study.

Considering the AUC values in the evaluation of solid nodules, the most effective model was the Herder model. The Mayo and Brock models followed respectively. The Mayo and Herder models yielded the highest AUC values here among all the groups evaluated in this study. This is because, as mentioned above, these two models, which are closely related, are likely to have been developed and validated in populations with high numbers of solid nodules. In addition, the AUC value for solid nodules with the Herder model is very close to the AUC value of the original study (0.92) [9]. This highlights the superiority of PET–CT for solid nodules.

As a result of various studies, many risk factors related to lung cancer were determined according to the clinical and demographic characteristics of the patients and the radiological characteristics of the nodules. However, these risk factors, and especially clinical and demographic factors, may differ in terms of their effects according to the structure of local populations and geographical features [10]. In the Brock model, female gender, family history of lung cancer, nodule type, localization of the nodule, and number of nodules are parameters that affect the probability of malignancy since there was a statistically significant difference between benign and malignant cases in the population investigated during the development of that model [7]. In the present study, a statistically significant relationship was found between male gender and malignancy and no other statistically significant differences were found between benign and malignant cases for the other parameters. Similarly, a history of smoking and a history of extrapulmonary malignancy at least 5 years ago were determined as risk factors in the Mayo and Herder models, but in our study, no statistically significant difference was found between benign and malignant cases for either parameter [8, 9]. Thus, the effects of these parameters on the differentiation of benign and malignant nodules in our study were reduced compared to the populations in the original studies. In such cases, it is inevitable that the performance of all three models will be decreased.

The main contribution of this study is its evaluation of nodules in the Turkish population with the currently used malignancy scoring systems by referring to definitive postoperative pathology results to retrospectively calibrate risk calculation models before using them in a new local population and provide a new optimal threshold value, as it mentioned in the literature [14].

The main limitation of the study is that it was conducted among patients who were followed in a thoracic surgery clinic in a reference center and operated on for pulmonary nodules. Most of the nodules in this study were already considered risky by clinicians and radiologists.

In conclusion, all models effectively differentiated benign from malignant pulmonary nodules in all groups except subcentimetric nodules and ground**-**glass nodules. However, none of the groups for which these models were effective had AUC values as high as those obtained in the original studies. This highlights the need to optimize models and malignancy risk thresholds for this population or develop a new model.

## Data Availability

Authors have all the data that used in this study. We can provide the data if requested.
